# Initial absolute monocyte count as an immune biomarker for clinical response in acute myeloid leukemia with monocytic differentiation

**DOI:** 10.1186/s43046-020-00044-2

**Published:** 2020-08-03

**Authors:** Ahmed Embaby, Ayman Fathy, Mohammad Al-Akkad, Ahmad Baraka, Taiseer Ibrahim, Nahla Zidan, Mohamed Refaat, Haitham Elsheikh

**Affiliations:** 1 Clinical Hematology Unit, Internal Medicine Department, Faculty of Medicine, Zagazig University, Zagazig, Al-Sharika 44519 Egypt; 2 Clinical Pathology Department, Faculty of Medicine, Zagazig University, Zagazig, Egypt; 3 Pathology Department, Faculty of Medicine, Zagazig University, Zagazig, Egypt; 4 Clinical Oncology Department, Faculty of Medicine, Zagazig University, Zagazig, Egypt

**Keywords:** Acute myeloid leukemia, Monocytic differentiation, Immune biomarker, Response, Absolute monocyte count

## Abstract

**Background:**

Absolute monocyte count (AMC) correlates with survival outcomes in various hematologic malignancies. However, its role in myeloid malignancies including AML needs to be highlighted. So, this prospective cohort study aimed to assess the effect of AMC on the treatment outcome and survival in a 56 adult de novo AML patients with monocytic differentiation, admitted to the Clinical Hematology Unit, Internal Medicine Department, in a tertiary referral hospital in Egypt, from July 2016 to June 2019.

**Results:**

The initial AMC was measured either by manual differential or the hematology automatic analyzer Sysmex XN-2000 and patients were classified by using receiver operating characteristic curve into two groups monocytopenic (≤ 4 × 10^9^/L) and non-monocytopenic (> 4 × 10^9^/L) group; including 24 (42.9%) and 32 (57.1%) patients, respectively. After a median follow up period of 7.7 (range 0.5–33.2) months, the monocytopenic group was associated with a significantly higher CR rate (*P* = 0.019), with a lower death as well as relapse and early relapse rates (*P* = 0.011, 0.033, and 0.002, respectively). Moreover, low initial AMC along with intensive induction were independently associated with complete response to induction chemotherapy with HR, 5.04 [1.37–18.58], *P* = 0.015, and 5.67 [1.48–21.71], *P* = 0.011, respectively by using the multivariate logistic regression model. Regarding survival, the monocytopenic group was associated with a better 3-year disease-free survival rate (*P* = 0.011) in univariate Cox regression only but did not reach significance in the multivariate model and did not affect the overall survival as well.

**Conclusion:**

Initial AMC was found to be an independent prognostic immune biomarker for treatment response in AML patients with monocytic differentiation. However, it did not appear as an independent predictor of survival in a multivariate analysis.

**Graphical abstract:**

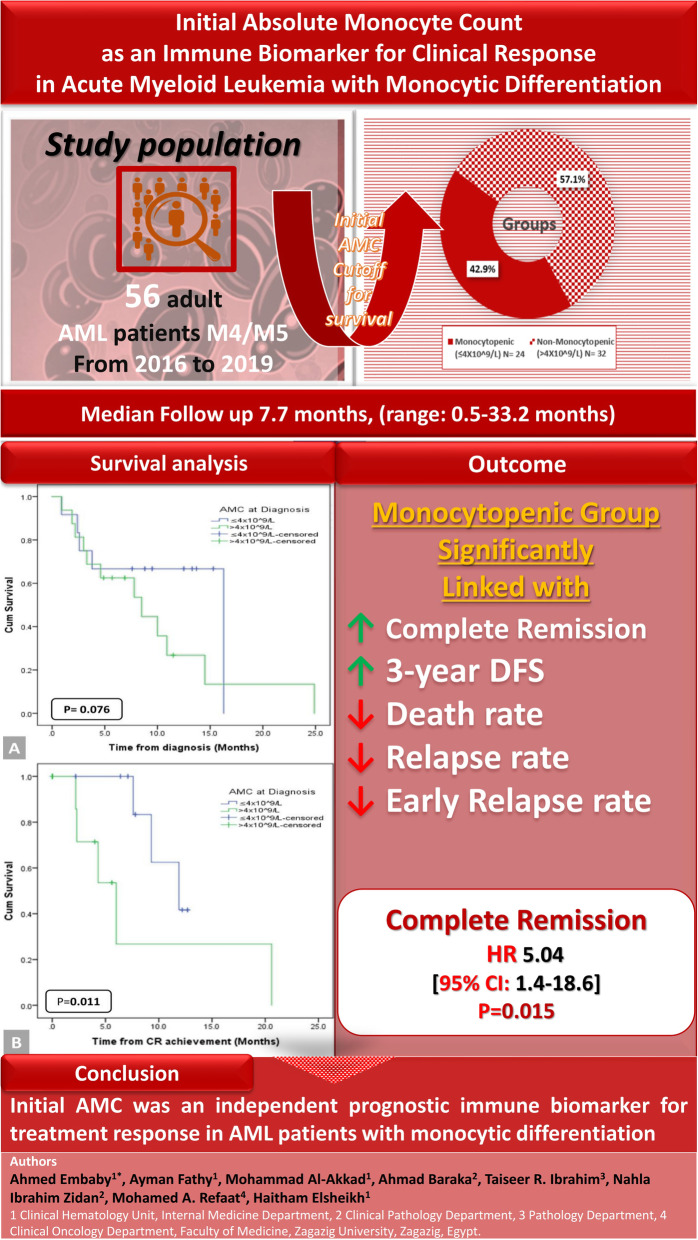

## Background

Acute myeloid leukemia (AML) is the most common acute leukemia in adults, accounting for approximately 78.3% of cases in this group, 1.2% of all cancers, and 34.7% of all leukemias; approximately 21,450 new cases of AML have been diagnosed annually in the USA [1].

The advent of novel therapies has significantly changed the AML outcome. However, AML remains largely incurable with a heterogeneous response to treatment [2]. Scant data are available on the contribution of the tumor microenvironment (TME) reflected in the circulating monocytes as a prognostic indicator for survival in AML [3]. Absolute monocyte count (AMC) correlates with survival outcome in lymphoma subtypes [4–6], multiple myeloma [7], and chronic lymphocytic leukemia [8]. Monocytes are key components of the innate immune system with dramatic increase with certain hematologic malignancies, particularly clonal monocytosis, and monocytic or myelomonocytic leukemia [9, 10]. Yet, their role needs to be defined in myeloid malignancies especially in AML with monocytic differentiation which includes French-American-British (FAB) M4 and M5 subtypes and shows distinct clinical features, such as the high risk of extramedullary disease, high leukocyte count, and coagulation abnormalities [11]. Accordingly, we assessed the effect of initial AMC on the treatment outcome and survival in AML patients with monocytic differentiation.

## Methods

### Patients

The study was designed to prospectively evaluate a cohort of 56 newly diagnosed adult patients having primary AML with monocytic differentiation with the exclusion of acute promyelocytic leukemia. All were 18-year-old or older with good performance status (ECOG-PS) [12], and treated at the Clinical Hematology Unit in a tertiary referral hospital in Egypt, from July 2016 to June 2019.

### Diagnosis and classification

The FAB classification [13], WHO 2016 criteria [14], and the International System for Human Cytogenetic Nomenclature [15] were the basis for AML diagnosis and subtyping using the morphological, immunophenotypic, and cytogenetic characteristics of leukemic blasts. In accordance with the Declaration of Helsinki, we collected written informed consent from each patient before starting the study with the agreement of our university Institutional Review Board.

### Data collection

We gathered patients’ clinical and laboratory data like age, gender, hemogram namely total leucocytic and monocytic counts, hemoglobin, platelet count, and the percentage of circulating and bone marrow (BM) blast cells; TLC and its differential including AMC were determined by hematology automatic analyzer Sysmex XN-2000 (Sysmex, Kobe, Japan) and confirmed by manual differential in cases of abnormal values (flagged values) or very low count that could not be detected by the device.

### Treatment plan

All patients were given induction chemotherapy by an anthracycline and cytarabine-based induction chemotherapy regimen; with high-intensity therapy, an induction 3 + 7 regimen consisting of continuous infusion cytarabine (100 mg/m^2^) daily for seven consecutive days combined with 3 days of doxorubicin (25 mg/m^2^). Elder patients were treated by low-intensity therapy; lower dose chemotherapy or hypomethylation agents. Patients who achieved complete remission received post-induction consolidation therapy which is comprised of three to four courses of high-dose cytarabine (1–2 g/m^2^ every 12 h on days 1, 3, and 5; total, 12 g/m^2^) [16].

### Criteria for therapy outcomes

Response to induction therapy was assessed after one or two courses of chemotherapy. Patients are considered to be in complete remission (CR) state if their BM blasts were below 5% along with evidence of the maturation of cell lines and normalization of peripheral blood (PB) counts and no evidence of extramedullary leukemia [17]. Primary induction failure (PIF) was defined as not achieving CR within two cycles of chemotherapy. Whereas, patients with early death (ED) were those who die in the first 30 days after initiating chemotherapy [18]. Hematological relapse was considered when more than 5% blasts were seen in BM aspirates or the appearance of extramedullary leukemia, while early relapse was considered if it occurred within 6 months of CR. Regarding survival, we calculated disease-free survival (DFS) from the CR date to the date of relapse or death, and overall survival (OS) from the initial diagnosis date to the time of death or last visit.

### Follow-up plan

After completion of consolidation, clinical and laboratory assessments including complete blood count (CBC), with blood smear, were done monthly for 2 years, then quarterly or biannually onward till the study came to an end. BM aspiration and biopsy only done if the peripheral smear is abnormal or cytopenias develop to rule-out relapse as recommended by the NCCN guidelines [19]. Patients who underwent allogeneic hematopoietic cell transplantation (HCT) were censored at the time of transplantation.

### Statistical analysis

A receiver operating characteristic (ROC) curve indicated that the sum of sensitivity and specificity reached a maximum for the value of AMC when 4 × 10^9^/L was used as a cutoff point for survival outcome as binary endpoints and subsequently this cutoff treated as a binary variable to group patients into monocytopenic (AMC ≤ 4 × 10^9^/L) or non-monocytopenic (AMC > 4 × 10^9^/L) groups. Shapiro test was applied to test data for normal distribution. Chi-square or Fisher’s exact tests were utilized to compare qualitative variables, while quantitative non-parametric variables were compared by the Mann–Whitney test. Spearman’s Rho test was used for linear correlation. Furthermore, independent predictors for the response were determined by enter multivariate logistic regression analysis model. Kaplan–Meier analyzer was used to calculate the OS and DFS with the log-rank test to compare survival curves. Patients undergoing allogeneic HCT were censored at the time of transplantation and the Cox-proportional hazards model was used for univariate and multivariate analysis. All tests were two-sided and a statistically significant difference was reached if *P* value ≤ 0.05. All statistical analyses were done using MedCalc Statistical Software (MedCalc16.4., Ostend, Belgium) and Statistical Package for Social Sciences (SPSS 20 Inc. Chicago, IL, USA).

## Results

**Fig. 1:**
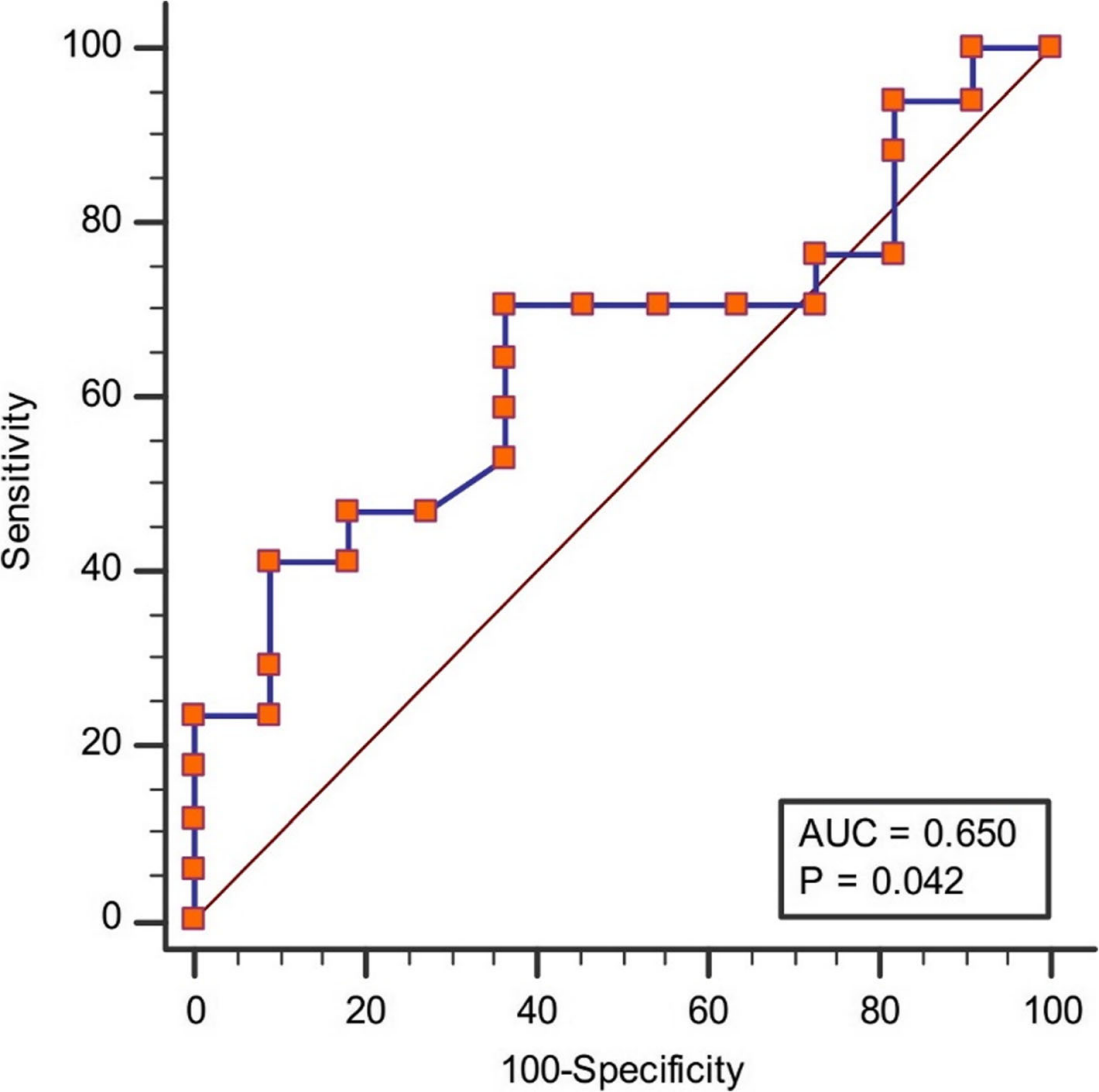
Initial AMC cut-off point: receiver operating characteristic curve (ROC) for initial AMC (absolute monocyte count) level for survival analysis, AMC at cutoff > 4 × 10^9^/L had an AUC of 0.65 (95% CI 0.511 to 0.772) with a sensitivity of 70.59% (95% CI 52.5–84.9%) and a specificity of 63.64% (95% CI 40.7–82.8%), *P* = 0.042

Fifty-Six previously untreated adult patients with de novo AML with monocytic differentiation were classified according to their initial AMC (Fig. 1) into 2 groups monocytopenic (≤ 4 × 10^9^/L) and non-monocytopenic (> 4 × 10^9^/L), including 24 (42.9%) and 32 (57.1%) patients, respectively (Table 1).

**Table 1:** Patients’ clinical characteristics and outcome [median (range) or *n* (%)] in both groups

Parameters		Groups		Total *N* = 56	*P* value
		Monocytopenic	Non-Monocytopenic		
		*N* = 24	*N* = 32		
Age, years		48 (20–61)	42.5 (19–75)	46 (19–75)	0.371
Age	≤ 60Y	22 (91.7%)	28 (87.5%)	50 (89.3%)	0.618
	> 60Y	2 (8.3%)	4 (12.5%)	6 (10.7%)	
Sex	Female	12 (50.0%)	16 (50.0%)	28 (50.0%)	1
	Male	12 (50.0%)	16 (50.0%)	28 (50.0%)	
PS	0–1	20 (83.3%)	26 (81.3%)	46 (82.1%)	0.84
	> 1	4 (16.7%)	6 (18.8%)	10 (17.9%)	
FAB subtype	M4	20 (83.3%)	20 (62.5%)	40 (71.4%)	0.088
	M5	4 (16.7%)	12 (37.5%)	16 (28.6%)	
Cytogenetic Risk	Failed	6 (25.0%)	8 (25.0%)	14 (25.0%)	0.183
	Favorable	2 (8.3%)	0 (0.0%)	2 (3.6%)	
	Intermediate	12 (50.0%)	22 (68.8%)	34 (60.7%)	
	Unfavorable	4 (16.7%)	2 (6.3%)	6 (10.7%)	
Intensive induction		16 (66.7%)	22 (68.8%)	38 (67.9%)	0.869
Initial TLC × 10^9^/L		6.8 (1.7–23)	50 (10–152.5)	20 (1.7–152.5)	< 0.001
Initial AMC × 10^9^/L		1.1 (0.1–4)	14.7 (5.1–35.1)	6.1 (0.1–35.1)	< 0.001
Initial HB g/dL		7 (5–11)	7.5 (4–10)	7 (4–11)	0.893
Initial PLT × 10^9^/L		25 (5–117)	42 (12–576)	33 (5–576)	0.014
Initial PB blast %		35 (0–88)	49.5 (13–94)	40 (0–94)	0.034
Initial BM blast %		54 (23–90)	56 (21–83)	55 (21–90)	0.504
Primary induction failure		8 (33.3%)	18 (56.3%)	26 (46.4%)	0.089
Early death		2(8.3%)	2(6.3%)	4 (7.1%)	0.765
Complete remission		18(75.0%)	14 (43.8%)	32 (57.1%)	0.019
Underwent HCT		10 (41.7%)	2(6.3%)	12 (21.4%)	0.001
Death		10(41.7%)	24 (75.0%)	34 (60.7%)	0.011
		*N* = 18	*N* = 14	Total *N* = 32	
Relapse^a^		6 (33.3%)	10 (71.4%)	16 (50.0%)	0.033
Early relapse^b^		0 (0.0%)	8 (80.0%)	8 (50.0%)	0.002

*PS* performance status, *FAB* French-American-British, *BM* bone marrow, *PB* peripheral blood, *TLC* total leucocytic count, *Hb* hemoglobin, *PLT* platelets, *HCT* hematopoietic cell transplantation

^a^Relapse calculated among patients who achieved CR

^b^Early relapse estimated among relapsed patients

### Baseline characteristics

**Fig. 2:**
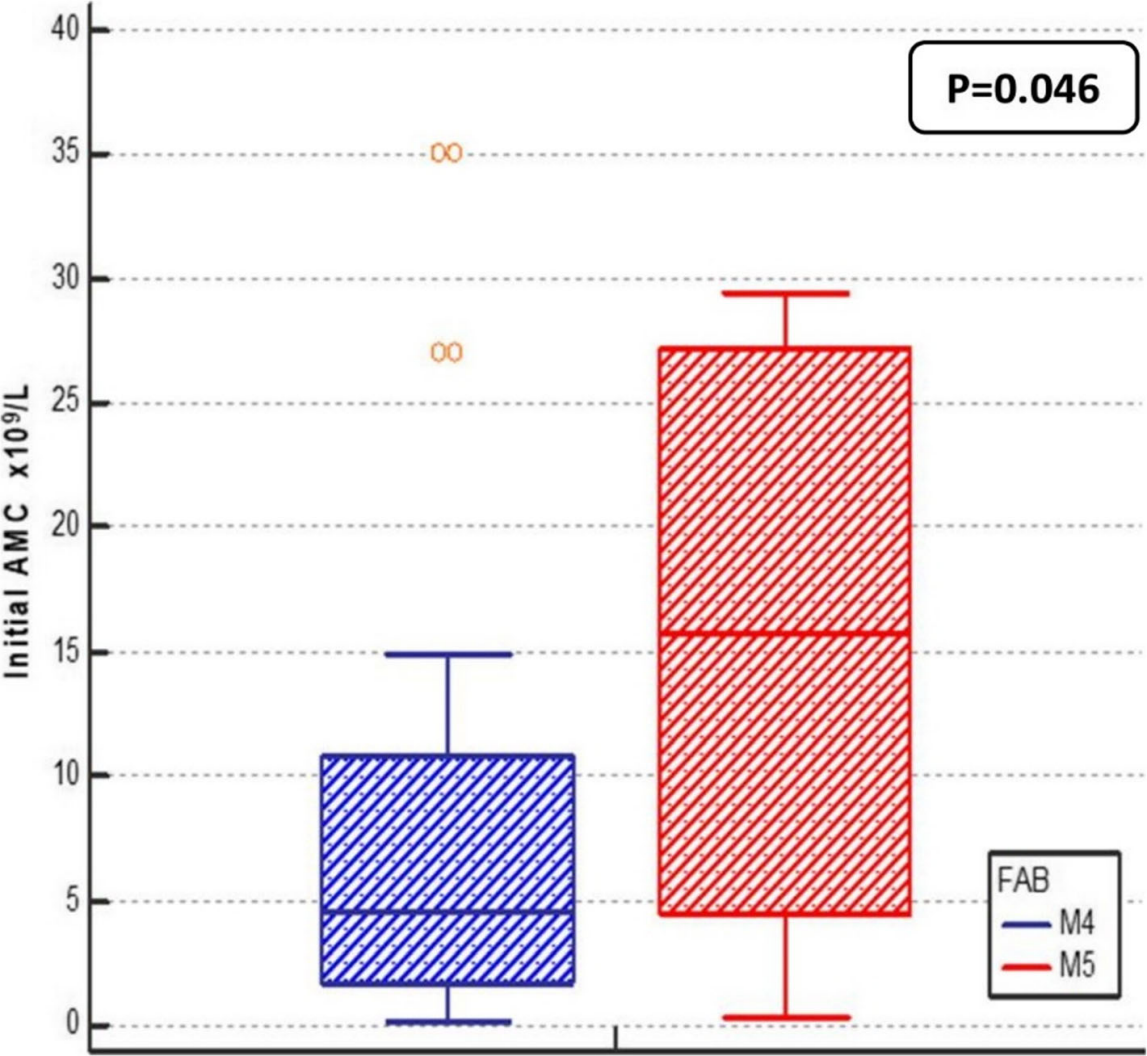
Comparison between initial AMC level in AML-subgroups: Box-plot diagram represents the range of initial AMC (absolute monocyte count) level as regard FAB subtype, *P* = 0.046; the upper and lower line in each box represents the 75th and 25th percentile respectively while the line through each box indicates the median. Whiskers represent the range between the minimum and maximum values excluding outliers (rounded markers)

**Fig. 3:**
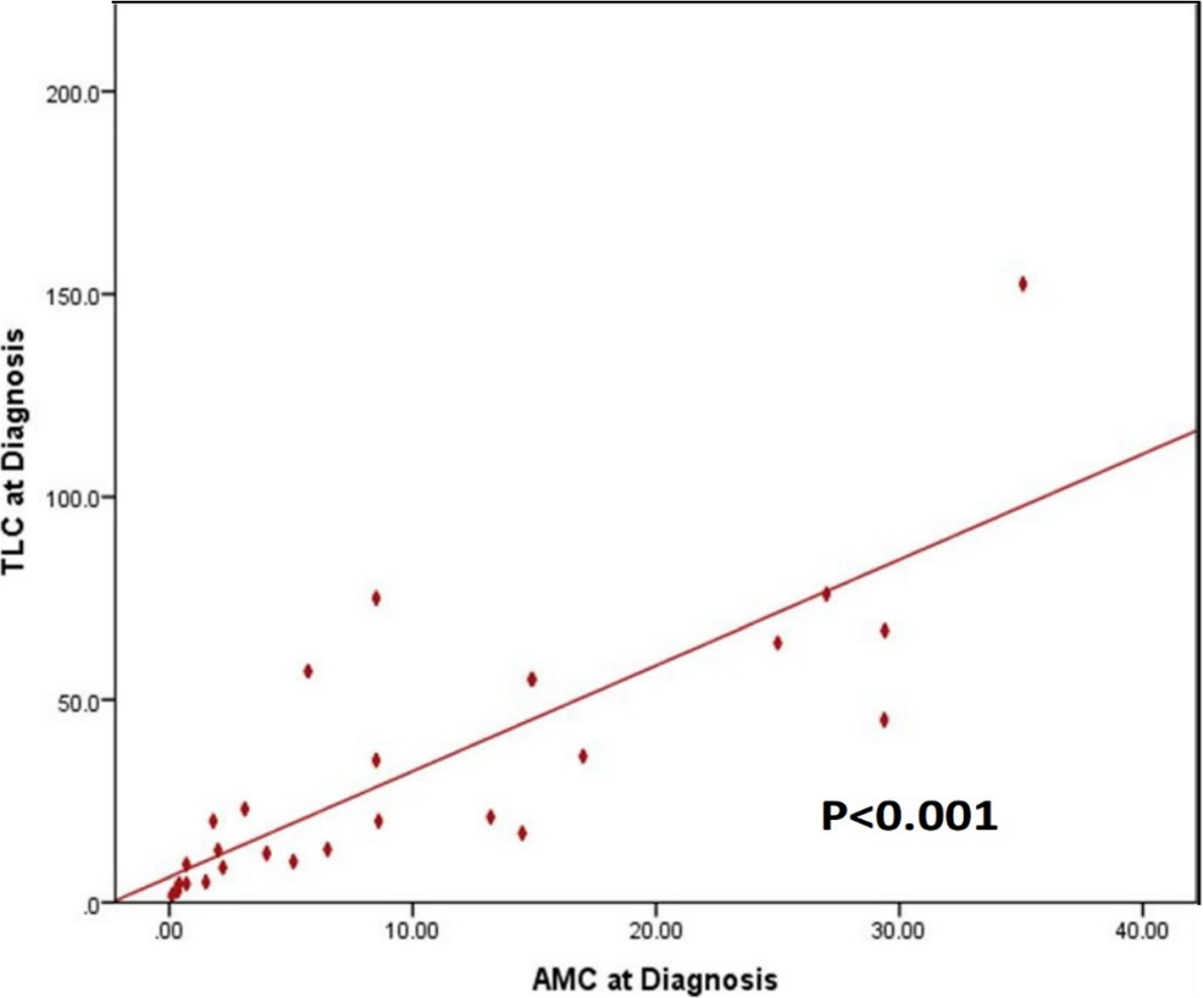
Linear correlation between the initial AMC (absolute monocyte count) and TLC (total leucocyte count)

The median age was 46 years (range, 19–75 years), with 50/56 (89.3%) patients were ≤ 60 years of age and 6/56 (10.7%) were > 60 years. Most of them had PS range from 0 to 1, 46/56 (82.1%) with male-to-female ratio of [1:1]. The median initial TLC of the entire cohort was 20 × 10^9^/L (range, 1.7–152.5 × 10^9^/L), 7 g/dL for hemoglobin [range, 4–11 g/dL], and 33 [5–576 × 10^9^/L] for platelets. Meanwhile, the median circulating PB blasts was 40% (range, 0 to 94%), while the BM blasts were 55% (range, 21–90%). The median initial AMC for all patients was 6.1 × 10^9^/L, with range [0.1–35.1 × 10^9^/L]. Regarding FAB classification, 40 cases were M4 (71.4%), with median initial AMC, 4.6 [range, 0.1–35.1 × 10^9^/L], and 16 (28.6%) were M5%, with median AMC, 15.8 [range, 0.4–29.4 × 10^9^/L], *P* = 0.046 (Fig. 2). Furthermore, a direct significant linear correlation was noticed between the initial AMC and TLC (*P* < 0.001), but not the other parameters (Fig. 3 and Table 2).

**Table 2:** correlation between the Initial AMCx10^9^/L and other studied parameters

Variable	Initial AMC × 10^9^/L	
	*r*	*P*
Age	− 0.001	0.997
PS	+ 0.119	0.388
Initial PB blast %	+ 0.202	0.16
Initial BM blast %	− 0.165	0.242
Initial TLC × 10^9^/L	+ 0.881	< 0.001
Initial Hb g/dL	− 0.096	0.482
Initial PLT × 10^9^/L	+ 0.16	0.24

*r* correlation coefficient, *AMC* absolute monocyte count, *PS* performance status, *BM* bone marrow, *PB* peripheral blood, *TLC* total leucocytic count, *Hb* hemoglobin, *PLT* platelets

Chromosomal analysis revealed that two (3.6%) patients had a favorable cytogenetic profile and 34 (60.7%) had an intermediate while six (10.7%) patients had unfavorable cytogenetics. Failure to attain cytogenetics was noticed in 14 (25.0%) patients. After initial workup, 38 (67.9%) patients received intensive induction chemotherapy and 18 (32.1%) patients were candidates only for less intensive chemotherapy. Moreover, the non-monocytopenic group was significantly associated with higher AMC, platelet (PLT) count, and PB blast (*P* < 0.001, *P* < 0.001, *P* = 0.014, and *P* = 0.034, respectively). However, no significant statistical difference was found with other variables (Table 1).

### Remission induction outcome

As shown in Table 1, 32 (57.1%) patients achieved CR, 26 (46.4%) patients did not achieve CR after at least two cycles of induction therapy (PIF), and four patients (7.1%) died early in the first 30 days of induction (early death) and by the end of follow-up, a total 34/56 (60.7%) of patients died. On following up the cases that achieved CR, 16/32 (50.0%) cases were relapsed, half of them relapsed within 6 months (ER), and 12 (21.4%) patients underwent HCT either after CR_1_ in those with high-risk cytogenetics or CR_2_ in relapsed patients. Moreover, the monocytopenic group was associated with significantly higher CR and HCT rates (*P* = 0.019 and 0.001, respectively), with a significantly lower death rate as well as relapse and early relapse rates (*P* = 0.011, 0.033 and 0.002, respectively). While no significant difference was found regarding PIF or ED.

### Response analysis

Binary logistic regression was performed to ascertain the effects of different variables on the likelihood that participants achieved CR and only the initial AMC and intensive induction therapy were independently associated with response to therapy in multivariate analysis with HR, 5.04 [1.37–18.58, *P* = 0.015] and 5.67 [1.48–21.71, *P* = 0.011], respectively (Table 3).

**Table 3:** Univariate and multivariable logistic regression analyses for response to therapy (achievement of CR)

Covariates	Univariate analysis		Multivariate analysis	
	OR (95% CI)	*P*	OR (95% CI)	*P*
Age (> 60Y vs. ≤ 60Y)	3.00 (0.50–17.95)	0.229		
Sex (Male vs. Female)	1.00 (0.35–2.88)	1		
PS (> 1 Y vs. ≤ 1)	2.33 (0.58–9.43)	0.234		
FAB (M4 vs. M5)	0.91 (0.29–2.82)	0.869		
Intensive induction (yes vs no)	4.33 (1.31–14.31)	0.016	5.67 (1.48–21.71)	0.011
Initial peripheral blast %	1.00 (0.98–1.02)	0.948		
Initial bone marrow blast %	1.00 (0.97–1.02)	0.779		
Initial TLC × 10^9^/L	1.02 (1.00–1.04)	0.069		
Monocytopenic (yes vs. no)	3.86 (1.21–12.28)	0.022	5.04 (1.37–18.58)	0.015
Cytogenetics (favorable vs others)	0.00 (0.0001–0.002)	0.999		

*PS* performance status, *FAB* French-American-British, *TLC* total leucocytic count, *OR* odds ratio, *95*% *CI* 95% confidence interval

### Survival analysis

**Fig. 4:**
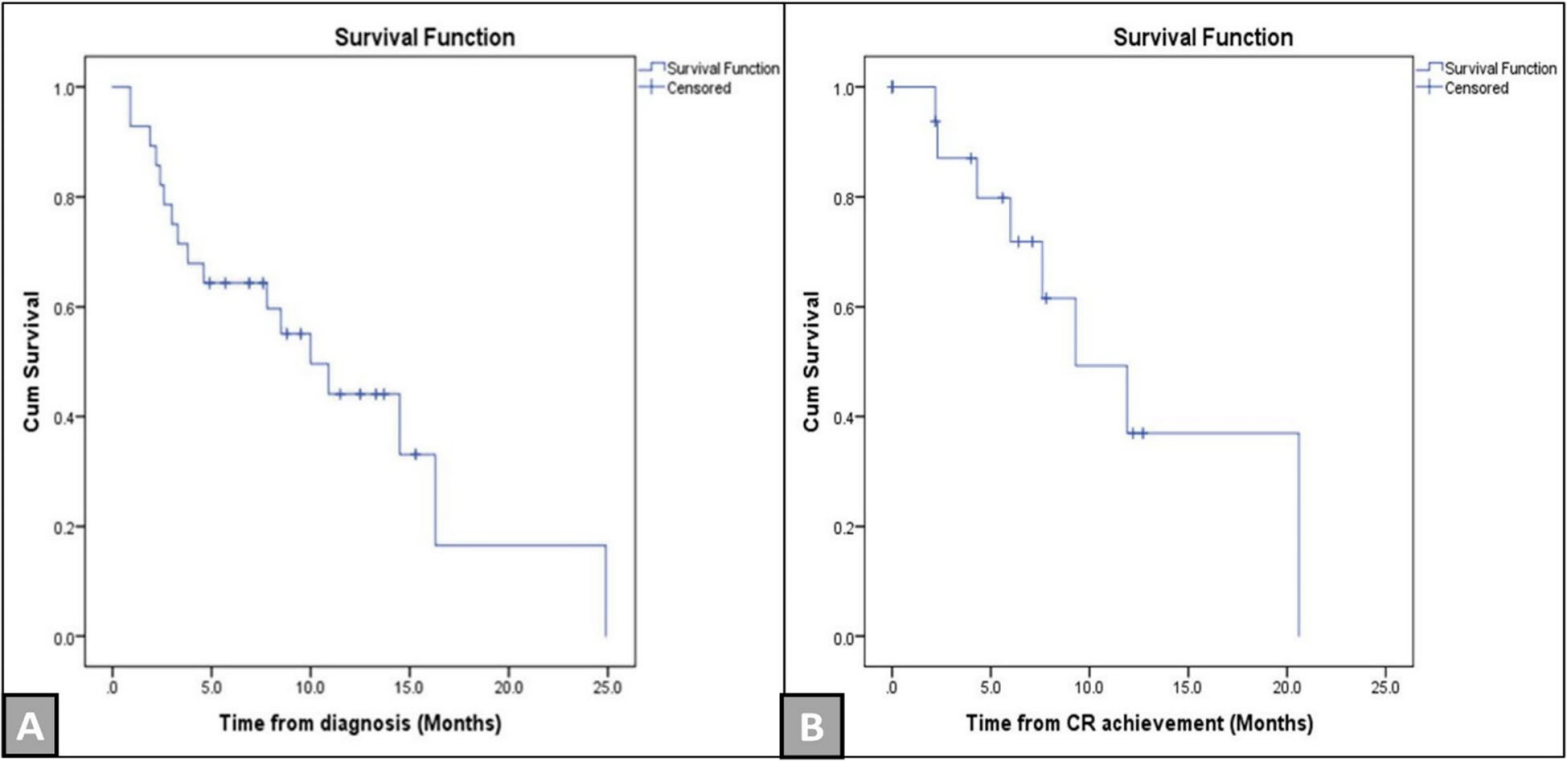
Survival analysis of the studied population. **a** The 3-year overall survival. **b** The 3-year disease-free survival

**Fig. 5:**
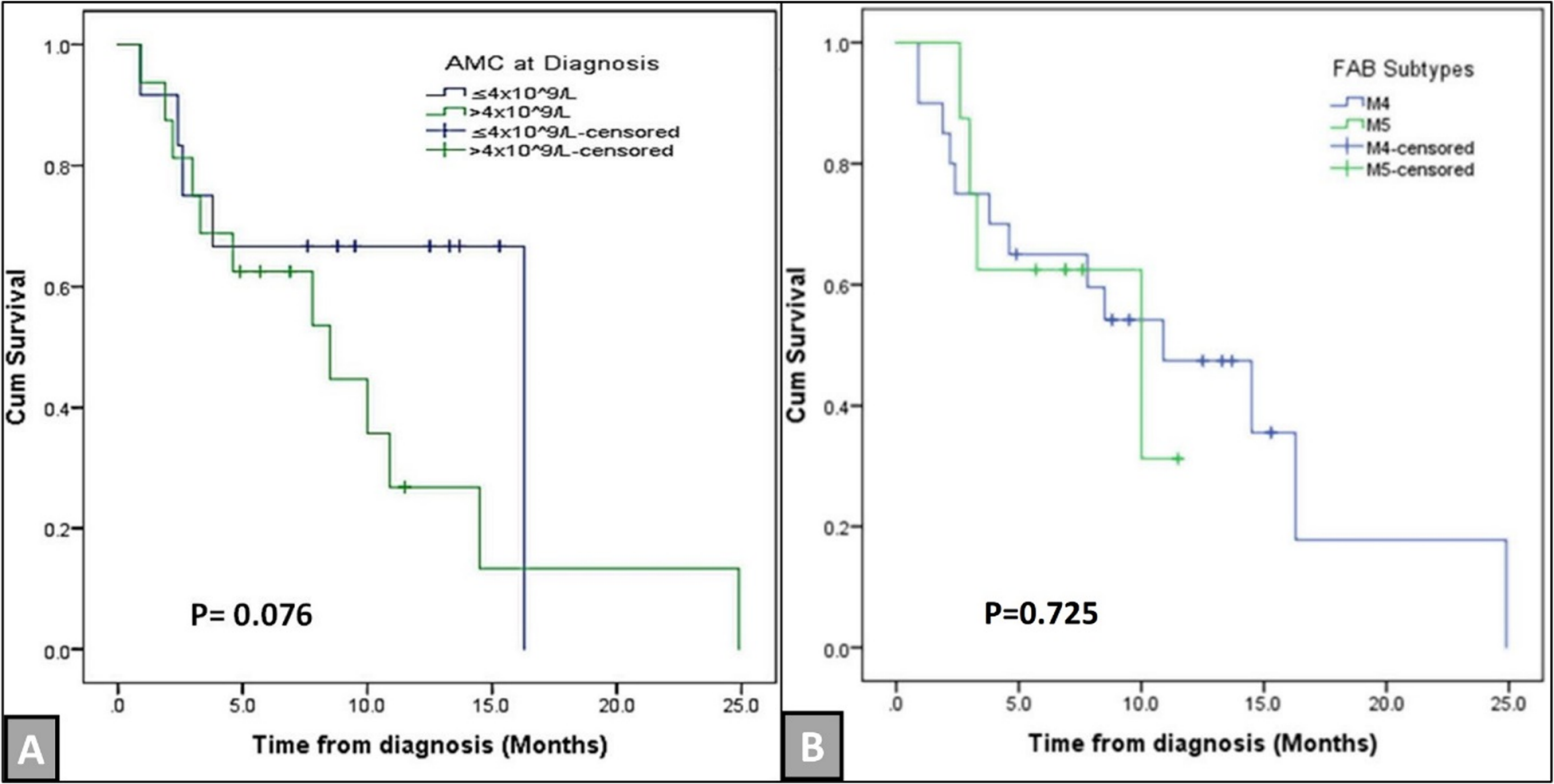
The 3-year overall survival analysis in different subgroups. **a** The 3-year overall survival as regard the initial AMC (absolute monocyte count) level. **b** The 3-year overall survival as regard the AML-subgroups

**Fig. 6:**
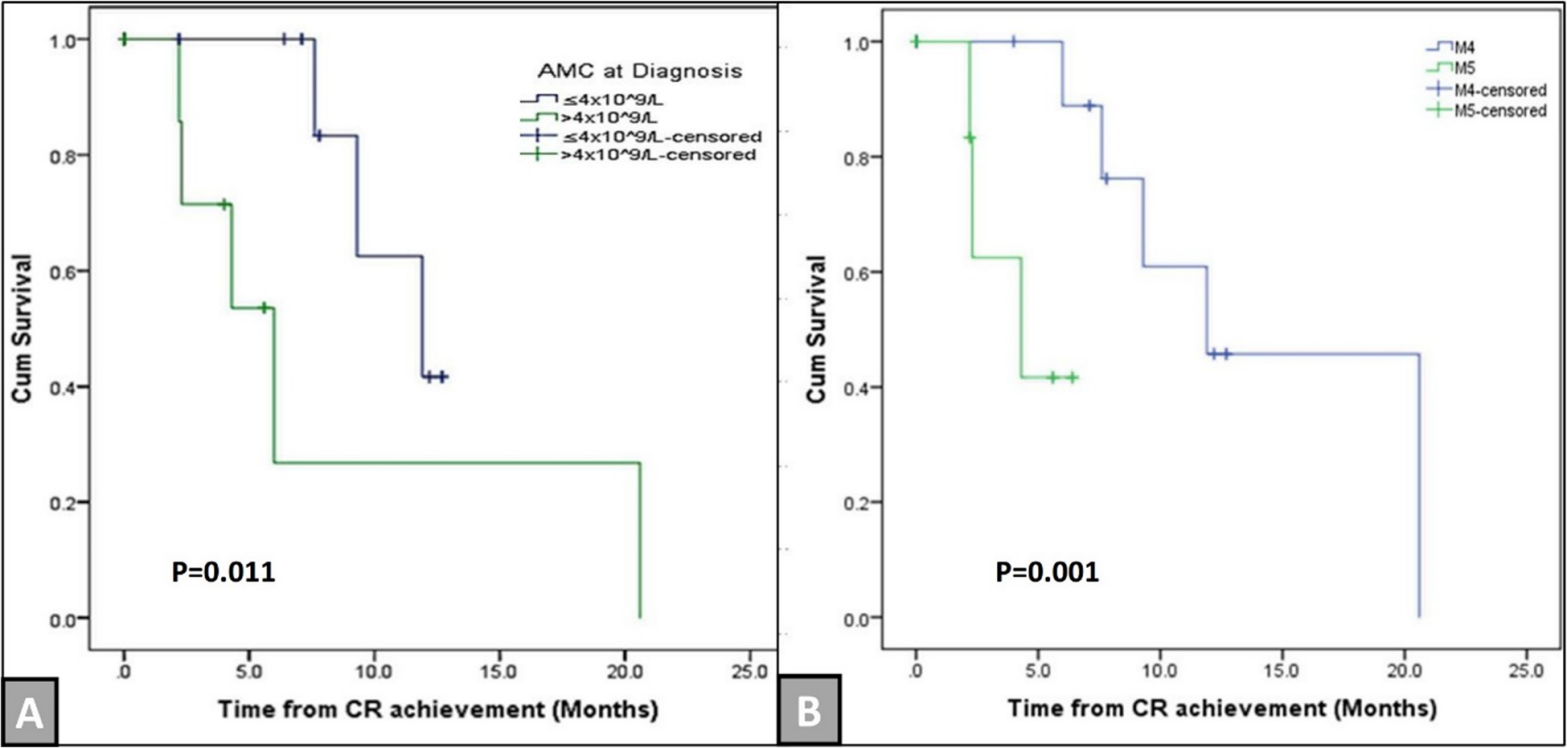
The 3-year disease-free survival analysis in different subgroups. **a** The 3-year disease-free survival as regard the initial AMC (absolute monocyte count) level. **b** The 3-year disease-free survival as regard the AML-subgroups

Patients were followed up for a median period of 7.7 months, range (0.5–33.2 months). The 3-year overall survival rate was 0.0% with a mean OS of 11.2 ± 1.3 months (95% CI 8.6–13.9 months) and the median was 10 ± 1.6 months (95% CI 6.9–13.1 months) while the 3-year disease-free survival rate was 0.0% with a mean of 12.1 ± 1.5 months (95% CI 9–15.1) and the median was 9.3 ± 1.9 months (95% CI 5.5–13.1) (Table 4 and Fig. 4a, b). Kaplan–Meier analysis showed no statistical difference in 3-year OS between the monocytopenic and non-monocytopenic group or M4 and M5 subtypes (*P* = 0.076 and 0.725, respectively) (Table 4 and Fig. 5a, b). Moreover, a statistical difference in 3-year DFS was found between both groups and M4 and M5 subgroups (*P* = 0.011, and 0.001, respectively) (Fig. 6a, b).

**Table 4:** The 3-year overall and Disease-free survival rates in both groups

Groups	Survival rate %	*P* value	Survival time, months			
			Mean		Median	
			Estimate ± SE	95% CI	Estimate ± SE	95% CI
The 3-years OS%						
Monocytopenic	0.00%	0.076	11.7 ± 1.4	8.9-14.4	16.3 ± 0	-
Non-monocytopenic	13.40%		9.6 ± 1.5	6.6-12.5	8.5 ± 2.1	4.3–12.7
Overall	0.00%		11.2 ± 1.3	8.6–13.9	10	6.9–13.1
The 3-years DFS%						
Monocytopenic	41.70%	0.011	11 ± 0.6	9.8–12.1	12 ± 1.9	8.1–15.7
Non-monocytopenic	0.00%		9 ± 2.5	3.5–13.6	6 ± 1.2	3.5–8.5
Overall	0.00%		12.1 ± 1.5	9–15.1	9.3 ± 1.9	5.5–13.1

*OS* overall survival, *DFS* disease-free survival, *SE* std. error, *95*% *CI* 95% confidence interval, *P* value of Log rank test

The Cox proportional hazards model evaluating the different variables affecting survival was summarized in Table 5 and showed that in univariate analysis, the following clinical parameters were significantly associated with OS, PIF, and initial platelet count (*P* < 0.001 and 0.002, respectively). However, in multivariate analysis, only PIF was independently associated with shorter OS (HR; 6.9 [2.8–17.8], *P* < 0.001). Regarding the DFS, initial AMC was found to have survival impact only in the univariate module (*P* = 0.021) but, it did not achieve significance in multivariate analysis.

**Table 5:** Univariate and multivariate Cox-regression analyses for overall and disease-free survival

Covariates	Overall survival				Disease-free survival			
	Univariate analysis		Multivariate analysis		Univariate analysis		Multivariate analysis	
	HR (95% CI)	*P*	HR (95% CI)	*P*	HR (95% CI)	*P*	HR (95% CI)	*P*
Age (> 60Y vs. ≤ 60Y)	0.80 (0.28–2.31)	0.682			0.04 (0.00–36.60)	0.35		
Sex (M vs. F)	1.29 (0.63–2.66)	0.485			1.89 (0.58–6.23)	0.293		
PS (> 1 Y vs. ≤ 1)	1.24 (0.50–3.04)	0.642			0.04 (0.00–23.31)	0.317		
PIF (yes vs. no)	7.8 (3.2–19.2)	< 0.001	6.9 (2.7–17.9)	< 0.001	2.5 (0.52–11.92)	0.296		
FAB (M4 vs. M5)	0.74 (0.36–1.52)	0.412			0.61 (0.19–1.98)	0.409		
Intensive induction (yes vs. no)	0.64 (0.31–1.32)	0.226			4.83 (0.99–23.59)	0.052	6.98 (0.69–70.77)	0.1
Initial PB blast %	1.00 (0.99–1.02)	0.552			1.02 (1.00–1.04)	0.055	1.05 (0.99–1.10)	0.092
Initial BM blast %	1.00 (0.99–1.02)	0.693			1.00 (0.98–1.03)	0.797		
Initial TLC × 10^9^/L	1.00 (0.99–1.01)	0.989			1.02 (1.00–1.04)	0.058		
Cytogenetics (favorable vs. others)	0.05 (0.00–56.25)	0.394			0.04 (0.00–36.60)	0.35		
Monocytopenic (yes vs. no)	1.91 (0.90–4.04)	0.091	1.03 (0.46–2.34)	0.941	3.65 (1.22–10.93)	0.021	1.26 (0.10–15.43)	0.856
Initial PLT × 10^9^/L	1.00 (1.00–1.01)	0.002	1.00 (0.99–1.04)	0.338	0.99 (0.97–1.01)	0.686		
Initial Hb g/dL	1.03 (0.85–1.25)	0.75			1.06 (0.77–1.47)	0.715		

*PS* performance status, *FAB* French-American-British, *PIF* primary induction failure, *BM* bone marrow, *PB* peripheral blood, *TLC* total leucocytic count, *Hb* hemoglobin, *PLT* platelets, *HR* hazard ratio, *95*%*CI* 95% confidence interval

## Discussion

An accurate prognostic assessment is fundamental to provide better clinical management and enhance the outcome of AML especially in an important subgroup like those with monocytic differentiation that carry high-risk features, including higher tumor burden represented in the brisk increase in leukocyte count, extramedullary disease, and coagulation abnormalities [11]. Furthermore, peripheral blood monocytes, the mirror of the immunosuppressive AML microenvironment [20, 21], have been tested as a prognostic factor for different types of hematological malignancies including AML [3–8]. However, that role was not clearly studied in M4/M5 patients. So, in our study, we shed some light on the significance of the initial AMC in a 56 cohort of newly diagnosed AML patients with monocytic differentiation.

To the best of our knowledge, we are the first prospective study to investigate the significance of the initial AMC in this subset of patients and our main findings revealed that in AML patients with monocytic differentiation, the monocytopenic group (defined by ROC curve as those with AMC level ≤ 4 × 10^9^/L) was significantly associated with higher CR rates, and lower death, relapse, and early relapse rates, with longer DFS.

Few published studies investigated the prognostic value of AMC in AML patients; one main study was done by Feng and coworkers, who found that high pretreatment AMC was associated with worse OS as compared with normal AMC patients [3]. This disagreement with our findings might be due to several factors like they used 0.8 × 10^9^/L as the cut-off point of AMC for survival outcome of AML patients obtained by the ROC curve. They included different FAB subtypes, unlike our study which focused on M4/M5 patients. Moreover, they carried out their study on Chinese patients, unlike ours which were all Egyptians, thus these racial/ethnic differences not only impact the AML survival rates but also the distinct monocyte behavior involving pro- and anti-tumor immunity, tumor advancement, and prognosis. In addition to that, they used a larger sample size with a longer follow up period, despite the retrospective design of the study that leads to selection bias.

Also, a recent study reported that high AMC was associated with lower CR rates, shorter OS, and leukemia-free survival in AML patients [22]. Despite having similar findings with our study, they were different in the retrospectively selected population that included all subtypes of AML together with M4/M5. Besides using different cut off value of the AMC. On the other hand, Bar and his colleagues reported that the pretreatment AMC was not associated with any statistically significant difference regarding OS or DFS rates [23]. This discrepancy with our findings might be owing to the inclusion of secondary AML along with all FAB subtypes and the retrospective selection of patients in remission in their study.

Remarkably in our M5 subgroup, higher initial AMC levels, as well as poor DFS rates were noticed compared to M4 subgroup, that could be explained by a different study that recognized higher TGF-β levels in M5 than M4 which play a physiologic role in monocyte maturation, differentiation, and recruitment in leukemia-associated macrophages (LAMS) with subsequent poor outcome [21, 24].

The intrinsic relationship between peripheral monocytes and clinical outcomes in patients with AML derived mainly from monocyte-derived mononuclear phagocytes like myeloid-derived suppressor cells (MDSCs), macrophages, and monocytes, the latter are vital constituent of the inflammatory response, might directly enhance malignant cell growth by the generation of numerous proinflammatory cytokines [25]. Besides, various laboratory-based researches have adopted the principle that tumor-associated macrophages (TAMs), evolved from peripheral blood monocytes, could promote systemic immunosuppression and significantly contribute to tumor cell invasion, migration, and angiogenesis [26]; even more, high AMC seems to be associated with an increased TAMs density [21]. Accordingly, an elevated AMC level may be a surrogate for higher monocytes and TAMs within the TME subsequently protect the AML cells and eventually carry a poor prognosis for those patients.

One of the limitations in our study is that our findings are derived solely from the CBC used in routine clinical practice, thus we are unable to determine phenotypic and functional changes within the monocyte-derived cell population that may have survival impact. So, we should be prudent when elucidating the results of this study, or probably our results might not be generalizable to other populations. Moreover, the prognostic role of the initial AMC as regard DFS was found only in the univariate analysis. Thus, it should be revalidated in the multivariate context including genetic information and monocyte subpopulations in the further prospective studies.

## Conclusions

This is the first prospective study to evaluate the relationship between the initial AMC and the outcome of AML patients with monocytic differentiation. Despite correlations of low AMC with DFS in univariate analysis, AMC did not appear as an independent predictor of survival outcome in multivariate analysis. Moreover, the initial AMC was found to be an independent prognostic factor for response to induction chemotherapy in those populations. Finally, before generalization of these findings, larger prospective multicenter studies considering racial and genetic information and monocyte subpopulations are required.

## Data Availability

All data analyzed and generated during this study are included in this published article.

## Abbreviations

AM: Absolute monocyte count
AML: Acute myeloid leukemia
BM: Bone marrow
CBC: Complete blood count
CI: Confidence interval
CR: Complete remission
DFS: Disease-free survival
ED: Early death
FAB: French-American-British
Hb: Hemoglobin
HCT: Hematopoietic cell transplantation
IL: Interleukin
LAMs: Leukemia-associated macrophages
MCP-1: Monocyte chemoattractant protein-1
MDSCs: Myeloid-derived suppressor cells
NCCN: National Comprehensive Cancer Network
NK cells: Natural killer cells
NR: No remission
OS: Overall survival
PB: Peripheral blood
PIF: Primary induction failure
PLT: Platelet
PS: Performance status
*r*: Correlation coefficient
ROC: Receiver operating characteristic
SPSS: Statistical Package for Social Sciences
TAMs: Tumor-associated macrophages
TLC: Total leucocytic count
TME: Tumor microenvironment
TNF-α: Tumor necrosis factor-alpha
VEGF: Vascular endothelial growth factor
WHO: World Health Organization
